# Helminth derived factors inhibit neutrophil extracellular trap formation and inflammation in bacterial peritonitis

**DOI:** 10.1038/s41598-021-92001-9

**Published:** 2021-06-16

**Authors:** Arun Chauhan, Atul Sharma, Jitendra K. Tripathi, Yuyang Sun, Pramod Sukumran, Brij B. Singh, Bibhuti B. Mishra, Jyotika Sharma

**Affiliations:** 1 Department of Biomedical Sciences, The University of North Dakota School of Medicine and Health Sciences, 1301 N Columbia Road, Grand Forks, ND 58202-9037 USA; 2 Department of Periodontics, School of Dentistry, University of Texas Health Science Center, San Antonio, TX USA; 3 Present Address: Department of Critical Care, Division of Anesthesiology, Critical Care and Pain Medicine, University of Texas MD Anderson Cancer Center, 1515 Holcombe Blvd, Unit 110, Houston, TX 77030-4009 USA; 4 Present Address: Department of Geriatrics, The University of North Dakota School of Medicine and Health Sciences, 1301 N Columbia Road, Grand Forks, ND 58202-9037 USA

**Keywords:** Cell biology, Immunology, Diseases, Pathogenesis

## Abstract

Despite their protective antimicrobial function, neutrophil extracellular traps (NETs) have been implicated in propagation of inflammatory responses in several disease conditions including sepsis. Highly diffusible exogenous ROS produced under such inflammatory conditions, can induce exuberant NETs, thus making inhibition of NETs desirable in inflammatory diseases. Here we report that helminth parasite excretory/secretory factors termed as parasitic ligands (PL) inhibit ROS-induced NETs by blocking the activation of nonselective calcium permeable channel Transient Receptor Potential Melastatin 2 (TRPM2). Therapeutic implication of PL mediated blockage of NET formation was tested in preclinical model of septic peritonitis, where PL treatment regulated neutrophil cell death modalities including NET formation and mitigated neutrophil mediated inflammatory response. This translated into improved survival and reduced systemic and local bacterial load in infected mice. Overall, our results posit PL as an important biological regulator of neutrophil functions with implications to a variety of inflammatory diseases including peritonitis.

## Introduction

Neutrophils are the “first responder” immune cells that are essential for combating microbial infection and their protective role in many infectious diseases has been described^1^. However, due to the highly non-specific and destructive nature of their intracellular contents, the turnover of neutrophils needs to be tightly regulated in order to avoid tissue injury and inflammation. A deregulation of this process resulting in persistent accumulation and overactivation of neutrophils has been implicated in neutrophil (PMN)-dependent inflammatory diseases^2, 3^. A recently established paradigm of neutrophil activation is the formation of neutrophil extracellular traps (NETs) which are decondensed chromatin fibrils coated with granular proteases and histones^4^. NETs can be triggered by pathogen-derived as well as host derived factors such as LPS, ROS, GM-CSF, IL-8, TNF-α, complement factor 5a and by contact with platelets^5^. Despite the advantageous properties of NETs in clearance of microbes, excessive NET formation has been implicated in many inflammatory diseases^6–10^. The modified histones and antimicrobial proteases decorating the NETs are thought to promote inflammation in these diseases by causing tissue injury and even generating immune responses to autoantigens. Thus, viable strategies to inhibit NET formation and the resulting hyperinflammation are highly desirable, a goal that has been met with little success so far.

Helminths are dubbed “master regulators” of immune response owing to their unique ability to establish chronic, yet asymptomatic infections in humans^11, 12^. The cestode *Taenia solium*, causative agent of Neurocysticercosis (NCC), induces immune suppressive effects which contribute to a long asymptomatic disease phase lasting 3–5 years^13^. The loss of these parasite-induced immune suppressive effects upon death of the parasite results in uncontrolled inflammation in the central nervous system (CNS) resulting in tissue pathology and disease severity^13, 14^. This ability of helminths to exert immunomodulatory effects has generated a strong interest in identification, characterization and use of their products as novel anti-inflammatory treatments for autoimmune and allergic diseases. We and others have reported immunosuppressive effects of helminth factors on myeloid cell maturation, influx and functions^15–18^. However, the effect of such factors on neutrophil functions, particularly NETosis is extremely understudied with only one report showing DNAse type activity of secreted factors from hookworm dismantling pre-formed NETs^19^. To the best of our knowledge, there are no reports yet of parasitic factors inhibiting the formation of NETs and the mechanism thereof. With the emerging role of exuberant NETs in propagating inflammatory response, the study of NET-modulatory effects of parasitic factors is timely and could present a viable therapeutic strategy for NET-mediated diseases.

In this study, we tested excretory/secretary factors (termed parasitic ligands, PL) of *Mesocestoides corti* (*M. corti*) a *Taenia solium* related cestode, most commonly used to model NCC in mice^20^, for their effect on oxidant-triggered and bacterial peritonitis-associated NETs. The beneficial modulatory effect of PL on neutrophil responses reported in this study will help design new therapies for septic peritonitis tested in current study, and other inflammatory diseases with underlying pathologic functions of neutrophils.

## Results

### PL abrogates ROS-induced TRPM2 activation and NET formation

**Figure 1:**
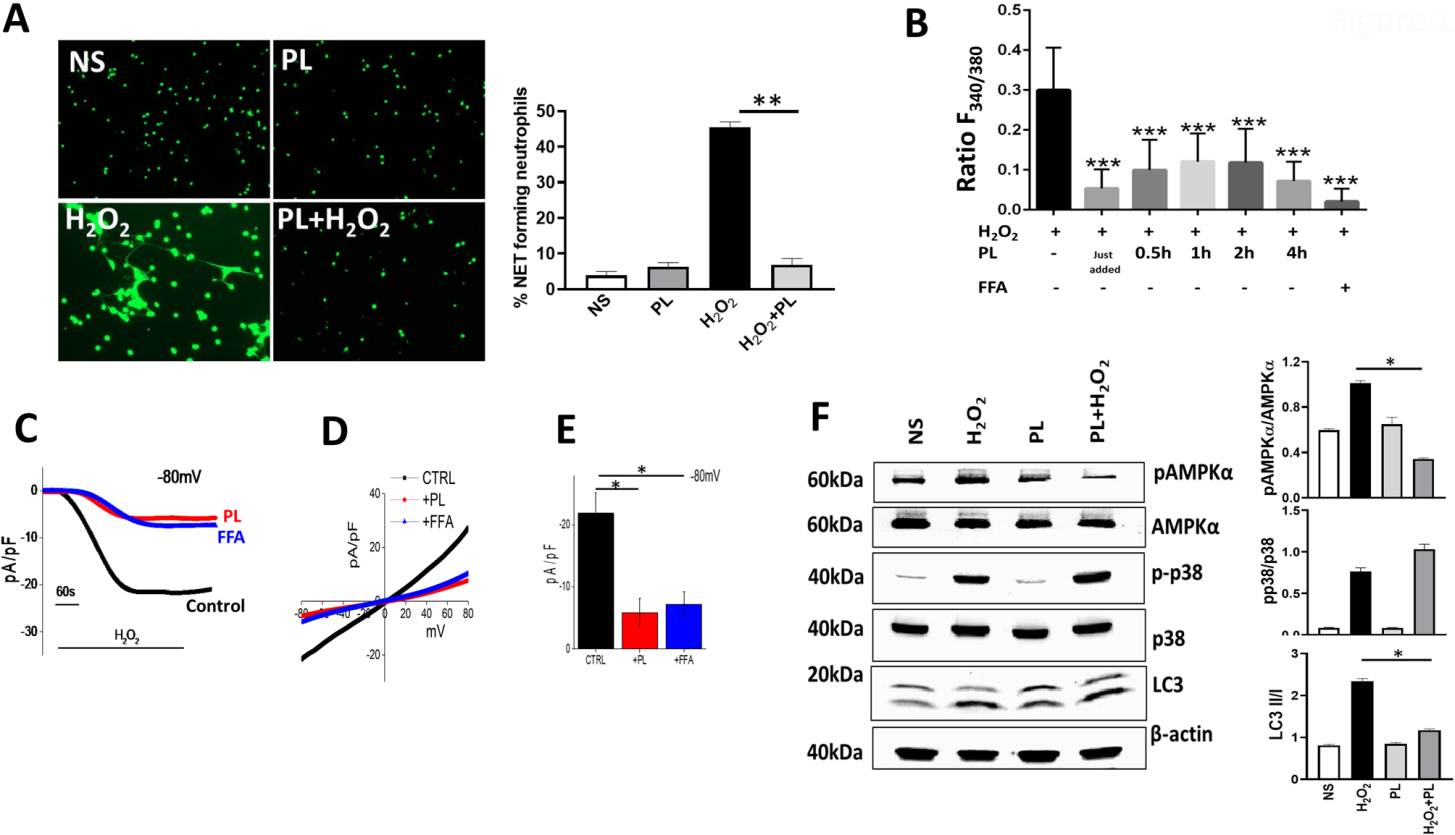
PL inhibits ROS induced TRPM2 activation and NET formation. (**A**) Peritoneal neutrophils were stimulated with 5 mM H_2_O_2_ for 4 h with or without PL pretreatment (25 µg/ml). Representative fluorescence images of Sytox Green stained NETs at magnification 200X are shown. Bar graph shows quantitation of NETs as average ± SE from 3 independent experiments. (**p < 0.01). (**B**) Ca^2+^ imaging was performed as described in methods in H_2_O_2_ stimulated neutrophils with and without 0.1 mM FFA or 25 µg/ml PL for indicated times. Bar graph shows quantification (mean ± SD) of the fluorescence ratio (F_340/380_) from an average of 40–60 cells in 3 independent experiments. (***p < 0.001). (**C**) The TRPM2 like current in H_2_O_2_ stimulated peritoneal neutrophils was measured by whole cell patch clamp method in the presence or absence of 0.1 mM FFA or 25 µg/ml PL as described in methods. Respective current–voltage (I-V) curves and current intensity (average of 8–10 recordings as bar graph) at − 80 mV are shown in (**D**,**E**). Statistically significant (*p < 0.05) attenuation of current density was found with PL and FFA treated neutrophils. (**F**) Western blot analysis was performed on equivalent protein amounts of cell lysates of neutrophils unstimulated (NS) or stimulated with H_2_O_2_ for 15 min with or without pretreatment (30 min) of 25 µg/ml PL, using antibodies against signaling proteins. Blots shown are representative of 3 independent experiments. Images of uncropped blots are provided in the supplementary material. Bar graph depicts densitometry analysis of protein band intensities expressed as ratio of indicated activated signaling protein to that of total signaling protein (*p < 0.05).

Oxidative stress is commonly associated with unbridled inflammation in sterile and infectious disease conditions; thus, we first tested the effect of helminth parasitic ligands (PL) on NET formation in response to ROS using an in-vitro method. As shown in Fig. 1A, unstimulated mouse primary neutrophils (NS) remained quiescent indicating that purification process did not induce any substantial NETs. Stimulation with H_2_O_2_ induced robust NET formation where 45 ± 1.5% of neutrophils exhibited NET formation as measured by Sytox Green staining (Fig. 1A). Importantly, treatment of neutrophils with PL 30 min prior to H_2_O_2_ stimulation significantly reduced the NET formation (Fig. 1A bar graph). Treatment with PL alone did not induce any NETs, suggesting that PL specifically modulates the activation status of neutrophils in response to H_2_O_2._

We have previously reported the involvement of intracellular calcium and cation channel TRPM2 in H_2_O_2_ induced NET formation^21^. We examined the effect of PL treatment on intracellular Ca^2+^ levels ([Ca^2+^]_i_) in H_2_O_2_ stimulated neutrophils. Addition of H_2_O_2_ significantly increased [Ca^2+^]_i_ levels (Fig. 1B, black bar). Immediate addition of PL caused a significant decrease in H_2_O_2_-induced Ca^2+^ influx compared to untreated control, which remained low in cells treated with PL for longer duration (0.5–4 h, Fig. 1B). The H_2_O_2_-induced Ca^2+^ influx was similarly inhibited by the addition of flufenamic acid (FFA), a specific inhibitor of cation channel TRPM2 used as a control in this assay. To examine this further, we assessed current recordings in whole cell configuration by patch-clamp analysis. Addition of H_2_O_2_ induced an inward current which was non-selective in nature and reversed between 0 and − 5 mV (Fig. 1C–E). Of note, I–V properties were similar to those characteristic of TRPM2 (Fig. 1D) and pretreatment of neutrophils with PL attenuated this H_2_O_2_-induced TRPM2 like current in primary neutrophils (Fig. 1C–E). This inhibitory effect of PL was similar to that observed with flufenamic acid (FFA), suggesting that PL likely exerts its inhibitory effect by blocking TRPM2 channels. Together, these results suggest that PL inhibits ROS-induced NET formation by blocking TRPM2 channel and Ca^2+^ entry.

We have previously reported the activation of a AMPK/p38 MAPK/autophagy pathway in neutrophils, which is required for NET formation in response to stimulation with H_2_O_2_^21^. We therefore sought examine if PL blocks this pathway to inhibit H_2_O_2_-stimulated NET formation. Western blot analysis of peritoneal neutrophils stimulated with H_2_O_2_ revealed an upregulation of phosphorylated AMPK, phosphorylated p38 as well as processed LC3 II indicative of autophagy activation (Fig. 1F), which is in line with our previous report^21^. Treatment of neutrophils with PL alone caused minimal changes in the levels of these signaling proteins. In contrast, pretreatment of neutrophils with PL 30 min. prior to H_2_O_2_ stimulation abrogated phosphorylation of AMPK but, had no impact on phosphorylated p38 MAPK (Fig. 1F). Interestingly, while PL did not downregulate the levels of processed LC3 II in H_2_O_2_ stimulated neutrophils, the ratio of processed LC3 II protein to nascent LC3 I was significantly reduced (Fig. 1F bar graph). This indicated a block in conversion of nascent autophagy protein LC3 I, also called ATG8, into processed form i.e. LC3-II. Overall, these findings revealed that PL inhibits NET formation in response to H_2_O_2_ stimulation by blocking TRPM2 activation and downstream AMPK and autophagy.

### PL treatment improves survival in septic peritonitis by regulating neutrophil responses

**Figure 2:**
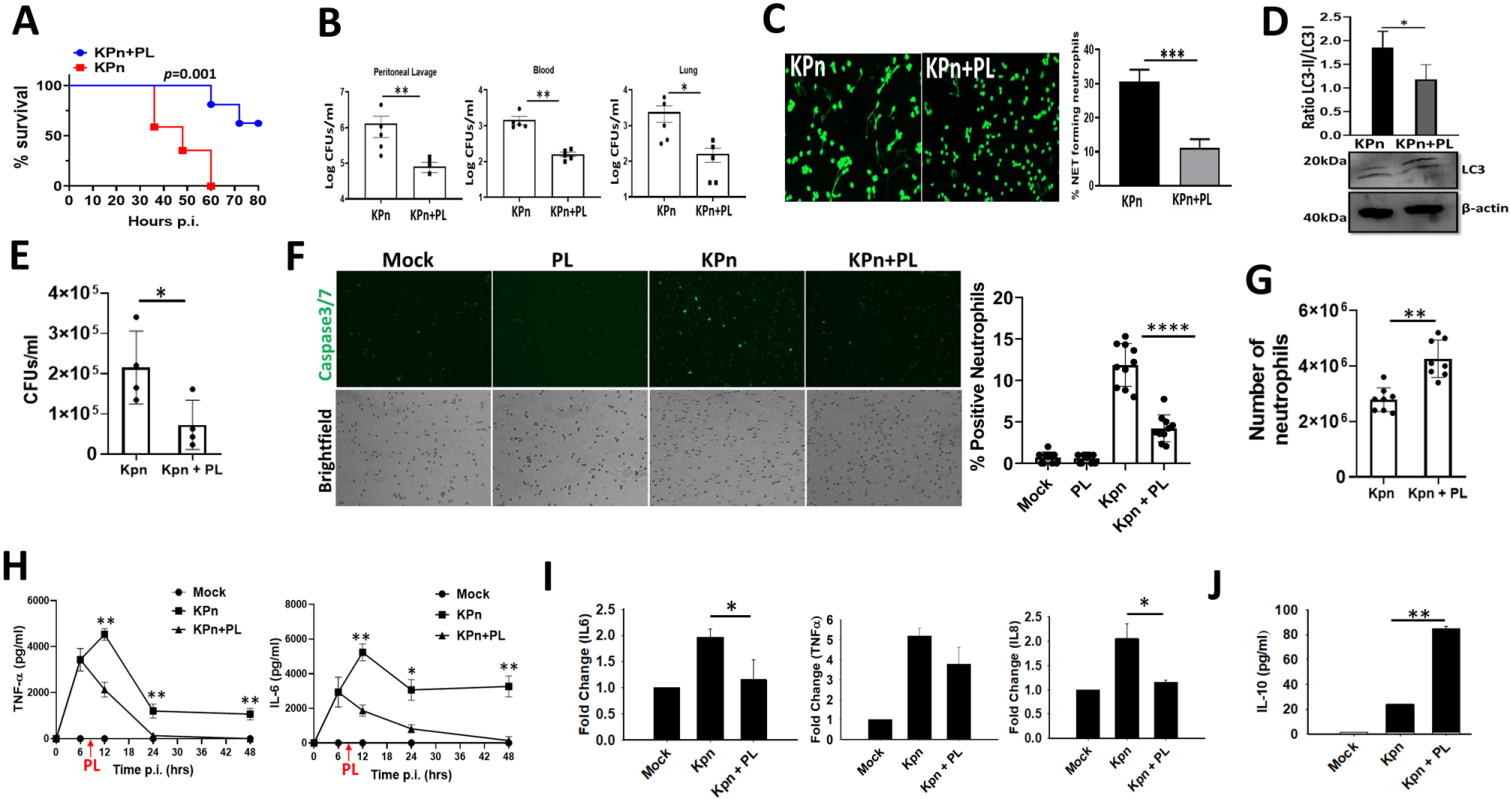
PL treatment improves murine septic peritonitis by regulating inflammatory response. (**A**) Survival of C57Bl/6 mice infected intraperitoneally with 5.0 × 10^3^ CFUs of KPn with or without PL treatment (20 mg/Kg injected intraperitoneally 8 h after infection). **p = 0.0015 by Kaplan–Meier log-rank test. *n* = 15 KPn and 17 KPn + PL in 3 independent experiments. (**B**) Bacterial load in peritoneal lavage, blood and lungs of mice at 24 h p.i. with KPn with or without PL treatment (20 mg/kg, 8 h p.i). (*p < 0.05; **p < 0.01). The experiment was repeated twice with 5 mice each time in each group. (**C**) Representative fluorescence images of the neutrophils isolated 24 h p.i. from peritoneal lavage fluid of mice infected with KPn with or without PL treatment (20 mg/kg, 8 h p.i.), and stained with Sytox Green to label NETs (green). Magnification 200X. The bar graph shows average ± SE of NETs from 4 independent experiments. (***p < 0.001). (**D**) Western blot analysis of autophagy in peritoneal neutrophils purified from mice infected with KPn with or without PL treatment. Uncropped blots are provided in the supplementary material. Bar graph depicts densitometry analysis of protein band intensities expressed as ratio of LC3-II to LC3-I protein. (**E**) Bacterial CFUs in neutrophils purified from peritoneal lavage of KPn infected mice isolated at 24 h p.i. with or without PL treatment. Magnetic column purified peritoneal neutrophils were washed twice with complete RPMI and lysed in sterile solution of 0.1% TritonX-100 before plating the serial dilutions on LB agar plates. The data shown is representative of three independent experiments with 4–5 mice in each group (*p < 0.05). (**F**) Representative images of cytocentrifuged neutrophils purified using magnetic columns as described in methods, from peritoneal lavage cells isolated at 24 h p.i. from mice infected with KPn with or without PL treatment. Neutrophils were processed for Caspse3/7 activation (green) by using CellEvent™ Caspase-3/7 Green Detection Reagent. Corresponding phase contrast images are shown to indicate the cell density in each sample. The bar graph depicts percentage of apoptotic neutrophils exhibiting active Caspase3/7 staining. Data from 5 mice in each group in 2 independent experiments is shown (****p < 0.0001). (**G**) Absolute numbers of neutrophils purified by positive selection from peritoneal lavage of PL treated and untreated KPn infected mice at 24 h p.i. is shown. Each dot represents neutrophil counts from one mouse (**p < 0.01). (**H**) Levels of Inflammatory cytokines were measured by ELISA in peritoneal lavage fluid at indicated times post-infection from mice infected intraperitoneally with KPn with or without PL treatment. Data is shown as average ± SE from 2 independent experiments with 3–4 mice in each group (**p < 0.01; ***p < 0.001). (**I**) Real Time PCR analysis of mRNA expression of inflammatory cytokines in peritoneal neutrophils purified as in (**D**). Data is shown as fold change in mRNA expression of IL-6, TNF-α and IL-1β over their respective baseline control level in neutrophils isolated from mock control animals (*p < 0.05). (**J**) Protein levels of IL-10 were measured in the culture supernatant of purified neutrophils incubated in complete RPMI for 2 h, using Legendplex MU Cytokine Release Syndrome Panel (13 Plex), following manufacturer’s instructions (BioLegend). Neutrophils were purified from peritoneal lavage isolated at 24 h p.i. from mock control (PBS alone) and KPN infected (with and without PL treatment).

Because oxidative stress and NETs are intimately involved in inflammatory exaggeration of septic peritonitis^22, 23^, we tested if PL treatment can provide survival advantage in preclinical model of this disorder. For this, mice were peritoneally infected with *K. pneumoniae* (KPn), which is one of the most common etiological agents of bacterial peritonitis^24, 25^. Mice undergoing septic peritonitis with KPn infection began exhibiting signs of overt disease by 36 h p.i. and became moribund by 48 h p.i. All mice succumbed to infection by 60 h p.i (Fig. 2A). In contrast, mice administered with a single therapeutic dose of PL 8 h after infection exhibited delayed appearance of disease signs with 65% mice surviving till 80 h p.i. In line with this improved outcome, significantly lower bacterial counts were recovered from the peritoneal lavage, lungs and blood of mice treated with PL, compared to the untreated, KPn infected mice (Fig. 2B). To understand the possible contributing mechanisms, various innate immune parameters were compared in mice undergoing KPn peritonitis with and without PL treatment. In line with previous reports, KPn infection induced substantial NET formation in the neutrophils infiltrating in the peritoneal cavity of mice upon KPn infection, where 31 ± 3.5% neutrophils produced NETs (Fig. 2C). In comparison, mice treated with PL displayed threefold reduction with only 11.1 ± 2.5% NETs (Fig. 2C bar graph). This downregulation of NET formation correlated with significantly reduced autophagy activation indicated by lower LC3-II/LC3-I ratio in neutrophils isolated from PL treated mice compared to those from untreated mice, undergoing KPn peritonitis (Fig. 2D). In order to understand the seemingly paradoxical finding of reduced NET formation but improved bacterial burden, we examined if PL treatment augmented intracellular bacterial killing in neutrophils. Indeed, significantly fewer bacteria were recovered from purified neutrophils isolated from peritoneal lavage fluid of PL treated mice, compared to their untreated counterparts, undergoing KPn peritonitis (Fig. 2E). In order to examine a possible neutrophil survival-enhancing effect of PL likely contributing to improved bacterial clearance, we compared apoptosis activation in neutrophils from septic mice with or without PL treatment. For this, activation of Caspase3/7 activation was analyzed in neutrophils purified from peritoneal exudates of mice. As shown in Fig. 2F, neutrophils from mock control or PL alone treated mice exhibited minimal staining of active Caspase3/7. Significantly higher frequency of neutrophils isolated from KPn infected mice harbored active Caspase3/7 at 24 h p.i. In comparison, mice receiving therapeutic treatment with PL exhibited much reduced Caspase3/7 activation in their neutrophils, indicating blockage of apoptosis upon PL treatment. This increased neutrophil survival correlated well with significantly higher numbers of neutrophils present in the peritoneal cavity of KPn infected mice upon PL treatment, as compared to those from infected but untreated mice (Fig. 2G). We next investigated the effect of PL on local and neutrophil-specific inflammatory response in septic peritonitis. Exorbitant amounts of TNF-alpha and IL-6 were seen in the peritoneal lavage of mice after KPn infection which peaked at 12 h p.i. in untreated mice (Fig. 2H). Although the levels of these cytokines dropped at 24 h p.i., their concentrations remained high even at 48 h p.i., a time when majority of surviving mice were moribund (Fig. 2A). In contrast, a significant reduction in the levels of inflammatory cytokines was observed within 4 h of injection of PL (12 h p.i., PL injection 8 h p.i.) indicating a potent immune modulatory effect of these parasitic factors on innate immunity. The levels of inflammatory cytokines continued to decrease in mice treated with PL and the levels were below detection limit by 48 h p.i. (Fig. 2H). A similar mitigation of inflammatory cytokine transcripts was observed in purified neutrophils from mice treated with PL (Fig. 2I). Interestingly, these neutrophils from PL treated mice produced significantly higher levels of immunosuppressive cytokine IL-10, compared to those from mice with KPn infection alone (Fig. 2J). Overall, our data shows a protective, immune modulatory effect of PL in septic peritonitis, where PL treatment improves neutrophils survival by preventing cell death by NETosis and apoptosis thereby improving the bacterial clearance while mitigating local and neutrophil-specific inflammation.

## Discussion

Excessive neutrophil activation and neutrophil extracellular traps can exacerbate inflammation in sepsis^23^. Here we report that helminth parasitic excretory/secretory factors (PL) can modulate these neutrophil responses. Specifically, we show that PL can inhibit NET formation in response to oxidant stimulation by blocking oxidant sensor channel TRPM2 and the downstream AMPK/autophagy axis. In a preclinical model of inflammatory septic peritonitis, therapeutic administration of PL abolishes NET formation and ensuing neutrophilic inflammation to improve bacterial clearance and overall disease outcome.

Helminths have long been known to release a plethora of immune modulatory factors that have co-evolved with mammalian immune system to facilitate establishment of chronic infections caused by these macroscopic parasites^26^. Long asymptomatic phase typically associated with these infections has given rise to the idea of “helminth therapy” oriented toward suppression of inflammation in hyperinflammatory immune diseases. While multiple excretory/secretory (ES) factors have been reported to exhibit immune modulatory properties, the mechanisms underlying their effect on specific cell types remain largely obscure, with most of the studies focusing on Th2 responses and macrophage polarization^27^. The recent recognition of neutrophils as major players in host immunity to helminth infections has brought into focus the role of NETs in anti-parasitic responses^28^. Indeed, *Nippostrongylus brasiliensis* was recently reported to produce DNase II as immune evasion mechanism to degrade NETs^19^. Dissolution of pre-formed NETs by DNAse treatment has been investigated in pre-clinical models of NET-mediated inflammatory diseases, however generation of multiple toxic byproducts including extracellular histones, which can paradoxically propagate inflammation^3^, makes this strategy clinically unsuitable. Our data presented here reports, for the first time, helminth factors that can inhibit the generation of NETs preemptively. While the identity of specific NET-inhibitory factor/s remains unknown at this time, our finding posits PL as immensely useful targets for use as adjuvant therapy in diseases with NET-mediated hyperinflammation.

Intra-abdominal infections are the second most common cause of sepsis, a life-threatening condition characterized by an overwhelming innate immune response^29^. Septic peritonitis occurs due to infectious insult of the peritoneal cavity causing exaggerated inflammatory responses, systemic spread of infection and eventually organ dysfunction^30^. NETs released during septic peritonitis are shown to activate coagulation cascade leading to microvascular hypoperfusion and end-organ damage^31^. Excessive production of highly diffusible ROS species during sepsis creates a highly oxidative environment^32^, which is conducive to exogenous ROS-mediated NET formation, as reported by us recently^21^. Cation channel TRPM2 acts as a sensor for ROS converting the oxidative stress into Ca^2+^ signaling^33–35^. TRPM2 is highly expressed on myeloid cells, and the adverse effect of oxidative stress during inflammation as seen in peritonitis may be due to over-activation of TRPM2. We have also previously reported inhibition of intracellular Ca^2+^ accumulation by parasitic factors via blockage of SOCE currents through both TRPC1 and ORAI1 Ca^2+^ channels on plasma membrane^16^. Similar to this activity, we found that PL inhibits H_2_O_2_ induced TRPM2 activation and intracellular calcium influx, which is required for H_2_O_2_-induced NET formation^21^. This regulation of TRPM2 activation and calcium influx by PL uncovers a novel regulatory mechanism of blockage of neutrophil activation pathway, which can clearly have implications for, not only for NET-mediated diseases but also for controlling pathophysiological consequences of oxidative stress seen in a variety of disease conditions.

Multiple signaling pathways of NET formation have now been identified which, depending upon the stimulus, can cross-talk with each other^36^. ROS themselves are regarded as signal-transduction factors capable of activating a number of signaling pathways^37^. We have previously shown that ROS sensing by TRPM2 activates AMPK/p38/autophagy pathway^21^. We found that PL treatment inhibits TRPM2 mediated calcium influx, AMPK phosphorylation and autophagy but exhibits no effect on p38 phosphorylation in response to H_2_O_2_. This is in line with our previous report showing no effect of helminth parasitic factors on p38 MAPK activation in macrophages^16^. Interestingly, p38 MAPK has been shown regulate the production of IL-10, an immunosuppressive cytokine commonly associated with chronic helminth infections^38, 39^. In light of these studies, lack of inhibition of p38 MAPK in neutrophils by PL is likely responsible for increased IL-10 secretion by neutrophils from PL treated mice that we observed in our study (Fig. 2J). In contrast to the widely reported modulation of MAPK signaling by helminths^40^, their effect on AMPK phosphorylation and autophagy activation is much less reported. Autophagy is a tightly regulated cellular process which has been intricately liked to NET formation, as evidenced by work from our and other laboratories^21, 41–43^. AMPK is a well-documented regulator of autophagy^44^ and we have shown that pharmacological inhibition of AMPK phosphorylation blocks autophagy and concomitant NET formation^21^. Thus, PL inhibition of AMPK phosphorylation is likely the mechanistic link to its blockage of autophagy and subsequent impediment of NET formation. However, contribution of additional players in this pathway that are specifically targeted by PL for NET inhibition cannot be ruled out. Because PL inhibits Ca^2+^ influx, one possibility is that it inhibits calcium-calmodulin dependent kinases, resulting in inhibition of downstream AMPK^45^. In addition, our finding that PL induced an instant decrease in Ca^2+^ currents, raises another possibility that PL directly binds to other plasma membrane cation channels and inhibits their function. Indeed, neutrophils abundantly express CRACs and several members of transient receptor potential (TRP) channel family that play a critical role in regulating neutrophil functions^46^. In addition, a role of calcium-activated potassium channel of small conductance (SK channel) in NET formation has been demonstrated^47^. It will be interesting to study the effect of PL on SK channel activation in neutrophils, an area to be investigated in future.

We found that PL treatment improved the disease outcome in mice undergoing septic peritonitis. This correlated with significantly reduced NET formation in the peritoneal cavity of PL treated mice. This suggests a deleterious inflammatory effect of NET in this disease, in line with a previous report^23^, which is supported by our data showing suppression of inflammatory cytokine production, but increased anti-inflammatory/regulatory cytokine IL-10 in these PL treated mice compared to untreated animals. PL treatment of septic mice also reduced the local bacterial burden as well as systemic spread of the infection. The improved bacterial clearance despite inhibition of NETs in PL treated mice suggested that PL may potentiate other antibacterial functions of neutrophils. Indeed, significantly lower intracellular bacterial loads were recovered in peritoneal neutrophils from PL treated mice compared to those from untreated animals. While macrophages reprogramming to enable antimicrobial function during bacterial and helminth co-infection has been documented^48^, ours is likely the first report showing parasitic factors-elicited potentiation of intracellular microbial killing in neutrophils.

Exuberant cell death due to hyperinflammatory response is a characteristic feature of sepsis, which results in depletion of cells required for clearance of pathogen. Mitigation of inflammatory cytokine response combined with inhibition of caspase3/7 activated apoptosis in neutrophils upon PL treatment suggests that PL prolonged the survival of neutrophils making them available for intracellular bacterial clearance. This is supported by our finding of significantly higher number of neutrophils present in peritoneal exudate of PL treated mice compared to those from their untreated counterparts. Whether these increased neutrophil numbers are also a result of increased influx in addition to reduced cell death by PL remains to be experimentally determined in our study. Nevertheless, increased neutrophil infiltration into the airway correlating with improved survival of mice co-infected with pulmonary bacterial and intestinal parasitic infection compared to bacterial infection alone has been reported^49^. PL mediated inhibition of caspase3/7 activation of apoptosis as well as NET formation in neutrophils raises an exciting possibility that it may act as a pan cell-death inhibitor interfering with a central node in programmed cell death pathway common to NETosis and apoptosis. In neutrophils, three cell death modalities pyroptosis, NETosis and apoptosis are thought to converge on activation of pore forming protein Gasdermin D^50^. Whether PL targets Gasdermin D remains to be experimentally determined. This may posit PL as an important therapeutic agent targeting gasdermin family of proteins, which are implicated in tissue damage and inflammation. Overall, our data strongly suggests a multipotent beneficial effect of PL on neutrophil functions and innate immune responses for improvement of disease outcome in preclinical septic peritonitis.

In summary, we have identified a novel NET inhibitory effect of helminth parasitic factors with protective implications in septic peritonitis, an acute inflammatory condition with underlying oxidative stress and excessive NET formation. The negative regulatory effect of PL on cation channel TRPM2 activation, Ca^2+^ influx and neutrophil cell death open up exciting areas of future research, which will posit these parasitic factors as valuable therapeutic targets to treat diseases caused by modulations in these processes.

### Experimental procedures

#### Bacterial strains and mice

The *K. pneumoniae* strain 43816 (KPn) was purchased from American Type Culture Collection. Bacterial stocks were prepared from bacteria grown to log phase in Luria–Bertani (LB) medium at 37 °C. All in vitro and in vivo experiments were performed using 6 to 8 weeks-old C57BL/6 bred in the animal facility of the University of North Dakota. The animals were used according to institutional and federal guidelines. All procedures and experimental protocols were approved by the Institutional Animal Care and Use Committee and Institutional Biosafety Committee at the University of North Dakota. The study is reported in accordance with ARRIVE guidelines.

### Isolation of parasitic ligands

Mesocestoides corti (*M. corti*) metacestodes were propagated in the peritoneal cavity of BALB/c mice by serial intraperitoneal infection^51^. Parasite ligands (PL) consisting of M. corti soluble factors were prepared from *M. corti* metacestodes by freezing and thawing as described by us^17^. PL was passed through 0.2 µ filter for sterilization prior to use.

### Analysis of NETs

For in vitro studies, peritoneal neutrophils were isolated using protocols published by us previously^42, 52^. Briefly, neutrophils were isolated from peritoneal lavage fluid at 8–12 h following injection with 4% w/v sterile thioglycollate solution. Purity was ensured by flow cytometry using CD11b and Ly6G antibodies (80–85% pure as assessed by flow cytometry). Neutrophils were stimulated with freshly prepared 5 mM H_2_O_2_ with or without PL (25 µg/ml) pretreatment (30 min) at 37 °C for 4 h. Cells were fixed with 4% paraformaldehyde and NETs were visualized by staining with fluorescent DNA dye Sytox Green (Molecular Probe) as described by us^42, 52, 53^. The percentage of NETs was manually calculated in a blinded fashion by dividing the number of NET-forming neutrophils with the total number of cells in 10 randomly selected microscopic fields and multiplying the values by 100. For the *in-vivo* NETs analysis, neutrophils were isolated from peritoneal lavage of mice at 24 h post KPn infection, neutrophils with or without PL treatment (20 mg/kg, 8 h post KPn infection), were cytocentrifuged on glass microscopic slides followed by Sytox Green staining and NET quantitation as described above.

### Electrophysiology

The patch clamp (electrophysiological) experiment was carried out using established protocol described by us^17, 54, 55^ in the tight-seal, whole-cell configuration at room temperature (22–25 °C) using an Axopatch 200B amplifier (Molecular Devices). Voltage ramps ranging from − 90 to 90 mV over a period of 1 s were imposed every 4 s from a holding potential of 0 mV and digitized at a rate of 1 kHz. A liquid junction potential of < 8 mV was not corrected, and capacitive currents and series resistance were determined and minimized. For analysis, the first ramp was used for leak subtraction for the subsequent current records. Currents were normalized to the initial size of the cell to obtain current densities (pA/pF).

### Measurement of calcium influx

The intracellular calcium levels were measured by fluorescence intensity using a ratiometric dye Fura-2 as previously described by us^17, 54, 55^. Briefly, primary neutrophils were loaded with 2 µm Fura-2 (Invitrogen) for 45 min followed by stimulation with H_2_O_2_ with or without pretreatment with PL for indicated times. Pretreatment with TRPM2 inhibitor 0.1 mM Flufenamic acid (FFA, Sigma Aldrich) for 30 min. was used as control. The maximum peak intensity upon addition of H_2_O_2_ in each condition in 40–60 individual cells was recorded with Compix (CCD camera-based imaging system). The images were processed with the C imaging, PCI software (Compix, Inc., Imaging Systems, Township Cranberry, PA, USA), to obtain ratios of Fura-2 fluorescence (F340/F380). The resulting values were plotted as bar graph.

### Western blot analysis

For the analysis of signaling pathway, neutrophils were plated in 90-mm dishes at the density of 10 × 10^6^ cells and stimulated with H_2_O_2_ for 15 min with or without pretreatment (30 min) of 25 µg/ml PL. Cells were lysed in 1X RIPA buffer and immunoreactivity of phospho-AMPKα1, AMPKα1, phospho-p38, p38, LC3 I/II, and β-actin antibodies (Cell Signaling Technology, Inc., USA) was detected by western blotting as described by us^21^. Densitometry of individual blots was done using ImageJ software (National Institutes of Health, Bethesda, Maryland, USA, https://imagej.nih.gov/ij/).

### Infection of mice and assessment of bacterial burden

Age and sex-matched wild-type C57BL/6 (WT) mice were injected intraperitoneal with 5000 CFUs of KPn in 200 µl of sterile PBS with or without PL treatment (20 mg/kg, 8 h post KPn infection). Mock mice received PBS only. Mice were monitored twice daily for signs of disease and mortality was recorded for up to 4 days post-infection (p.i.). In some experiments, the mice were euthanized at indicated times post-infection and blood, lungs and peritoneal lavage were harvested and processed for bacterial burden analysis as described by us^52, 53^. To examine total number of neutrophils infiltrating the peritoneal cavity, peritoneal lavage fluid collected at 24 h p.i. was passed through magnetic column (Miltenyi Anti LY6G microbeads) for positive selection and absolute neutrophil count was determined using hematocytometer. The cells were washed twice with complete RPMI followed by lysis in 0.1% TritonX-100. Lysates were serially diluted and plated on LB agar plates. Colony forming units were determined after 24 h incubation at 37 °C.

### Analysis of Caspase3/7 activation

Mice were infected intraperitoneally with KPn with or without PL treatment (20 mg/kg, 8 h post infection). At 24 h p.i., neutrophils were purified from peritoneal lavage fluid using magnetic column (Miltenyi Anti LY6G microbeads) and loaded with optimized concentration of CellEvent Caspase-3/7 Green Detection Reagent (Invitrogen) following manufacturer’s instructions. Cells were then analyzed for Caspase3/7 activation by imaging using EVOS FL microscopy under 200× magnification. The percentage of apoptotic neutrophils was manually calculated in a blinded fashion by dividing the number of Caspase3/7 positive neutrophils with the total number of cells in 10 randomly selected microscopic fields and multiplying the values by 100.

### Cytokine analysis

Peritoneal lavage fluid collected at indicated times from mice infected with KPn with or without PL treatment was used to measure inflammatory cytokines TNF-α and IL-6 by enzyme-linked immunosorbent assay by following manufacturer’s instructions (BD OptEIA; BD Biosciences).

For transcript analysis, total RNA was isolated from magnetic column purified peritoneal neutrophils from mock (PBS), and KPn infected mice with or without PL treatment and processed for q-RT PCR as described by us^56^ Primers used included: 18 S (sense) 5′-CATGTGGTGTTGAGG AAAGCA-3′ and (antisense) 5′-GTCGTGGGTTCTGCATGATG-3′; IL6 (sense) 5′-TTCATCCAGTTGCCTTCTTG-3′ and (antisense) 5′-GGGAGTGGTATCCTCTGTGAAGTC-3′, TNFα (sense) 5′-GGGTGTTCATCCATTCTCTACC-3′ and (antisense) 5′-TTGGACCCTGAGCCATAATC-3′ and IL8 (sense) 5′-CCTGCTCTGTCACCGATG-3′ and (antisense) 5′-CAGGGCAAAGAACAGGTCAG-3′. Protein levels of IL-10 were determined by purifying neutrophils from peritoneal lavage of mock (PBS), and KPn infected mice with or without PL treatment followed by incubation at 37 °C for 2 h in Complete RPMI (Gibco). Culture supernatants were briefly centrifuged and analyzed using Legendplex MU Cytokine Release Syndrome Panel (13 Plex), following manufacturer’s instructions (BioLegend).

### Statistical analysis

Statistical analysis of survival studies was performed by a Kaplan–Meier log-rank test. The Student’s t test and one-way analysis of variance was used for comparison of mean values in different groups [SigmaPlot 10.0 software (Systat Software, San Jose, CA, USA)]. A *p* value < 0.05 was considered to be statistically significant.

### Disclaimer

The content is solely the responsibility of the authors and does not necessarily represent the official views of the National Institutes of Health.

## Supplementary Information

Supplementary Information 1.
