# Supplementary figures and images for: Helminth derived factors inhibit neutrophil extracellular trap formation and inflammation in bacterial peritonitis
Source: Sci Rep. 2021 Jun 16;11:12718. doi: 10.1038/s41598-021-92001-9 (PMC8209178; doi:10.1038/s41598-021-92001-9)

Fig. 1F

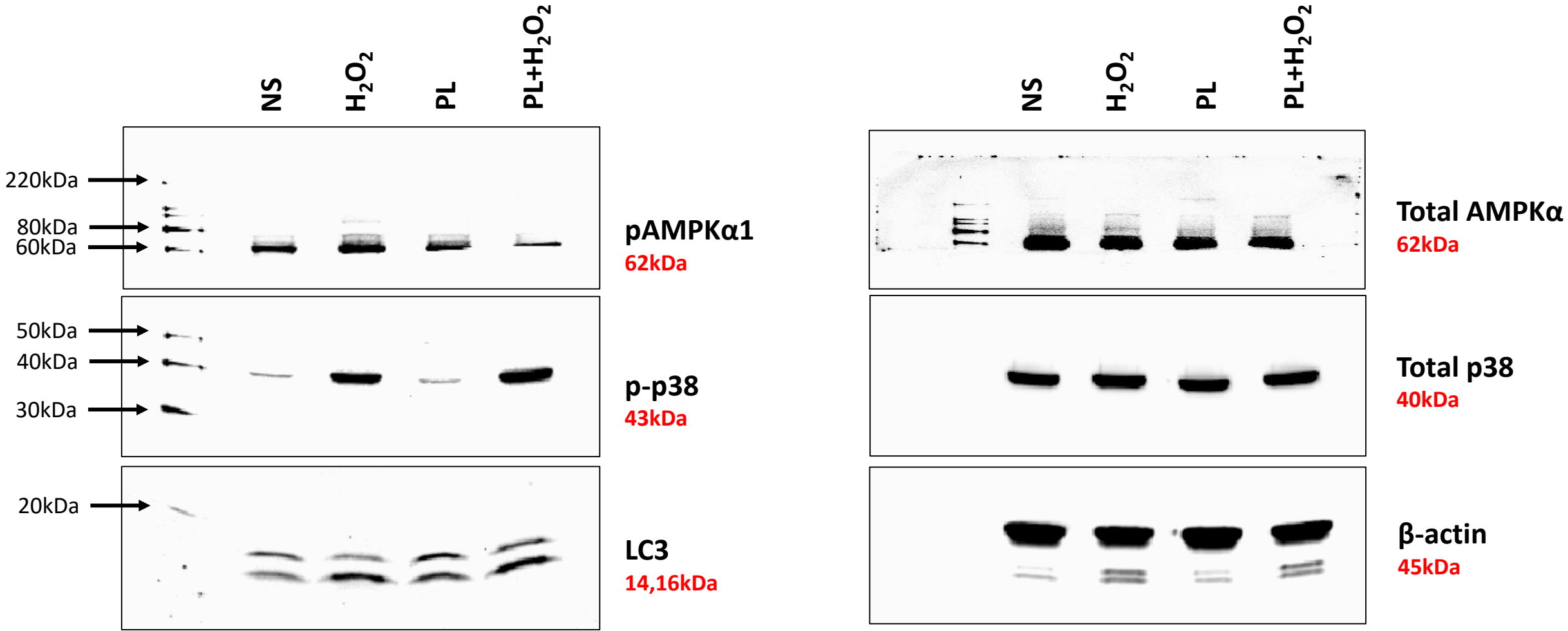

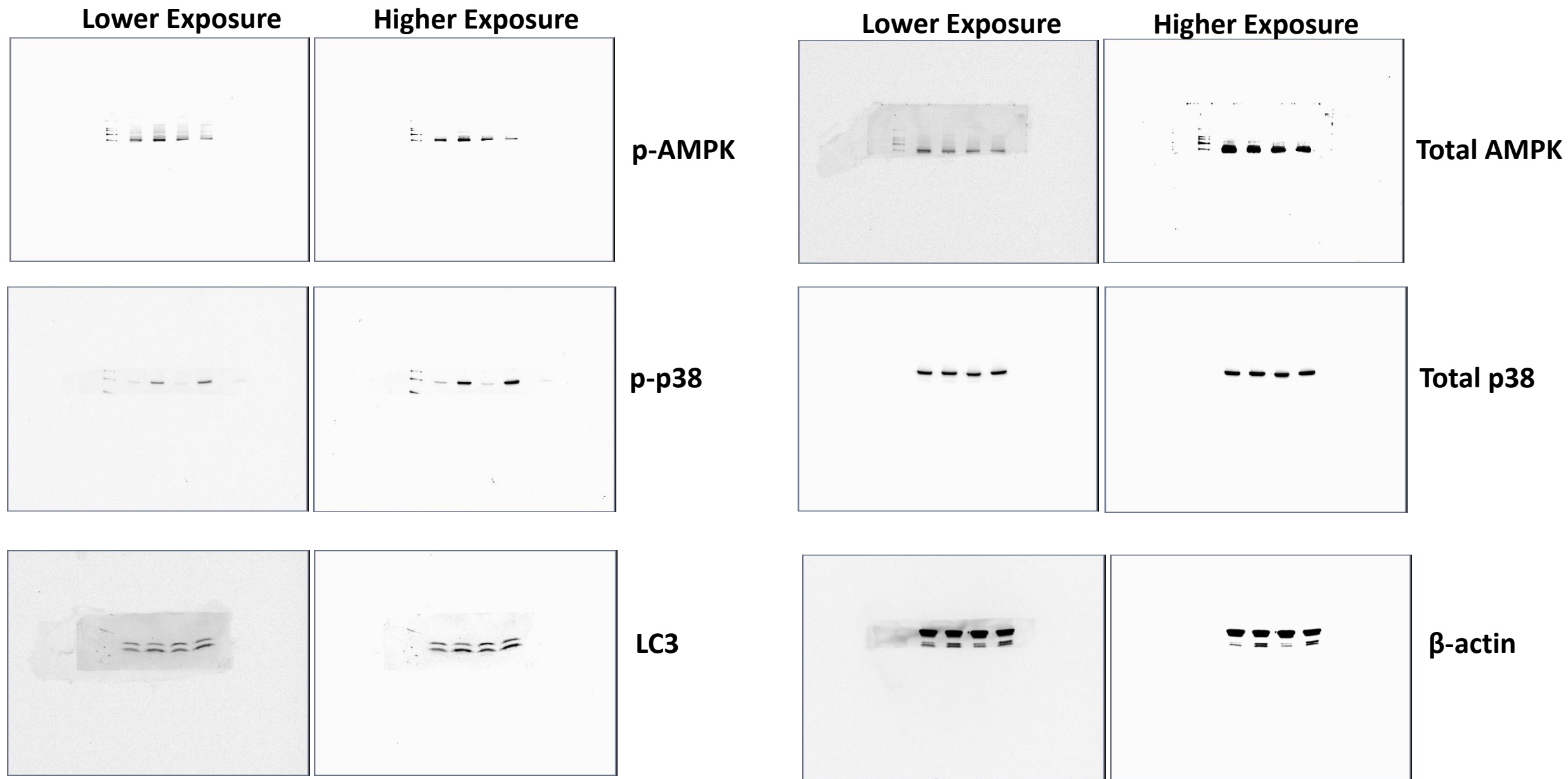

**Fig. 1F Multiple Exposures**

Fig. 2D

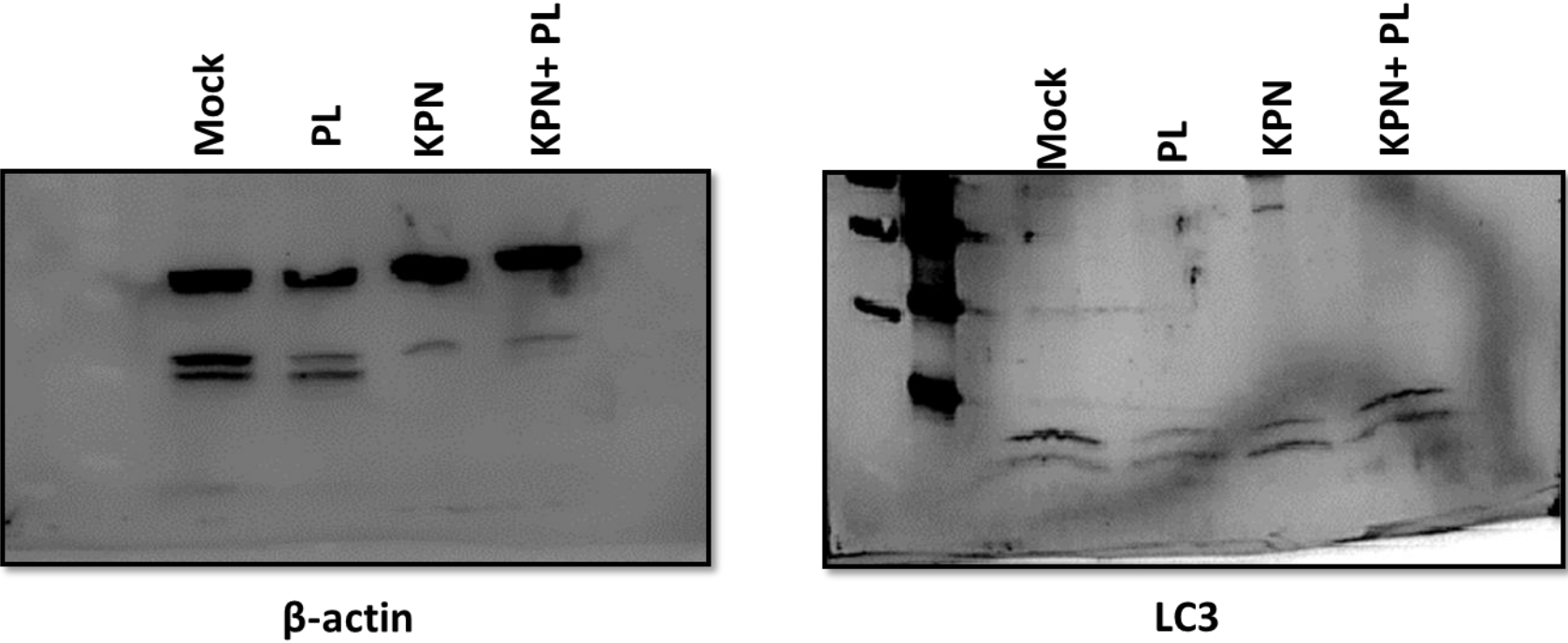

Supplement: Supplementary file 1 — Supplementary Information 1. [file 41598_2021_92001_MOESM1_ESM.pdf]
